# Human Adipose Derived Stromal Cells Heal Critical Size Mouse Calvarial Defects

**DOI:** 10.1371/journal.pone.0011177

**Published:** 2010-06-17

**Authors:** Benjamin Levi, Aaron W. James, Emily R. Nelson, Dean Vistnes, Benjamin Wu, Min Lee, Ankur Gupta, Michael T. Longaker

**Affiliations:** 1 Hagey Pediatric Regenerative Research Laboratory, Plastic and Reconstructive Surgery Division, Department of Surgery, Stanford University School of Medicine, Stanford, California, United States of America; 2 Division of Advanced Prosthodontics, Biomaterials, and Hospital Dentistry, School of Medicine, University of California Los Angeles, Los Angeles, California, United States of America; Istituto Dermopatico dell'Immacolata, Italy

## Abstract

**Background:**

Human adipose-derived stromal cells (hASCs) represent a multipotent cell stromal cell type with proven capacity to differentiate along an osteogenic lineage. This suggests that they may be used to heal defects of the craniofacial or appendicular skeleton. We sought to substantiate the use of undifferentiated hASCs in the regeneration of a non-healing mouse skeletal defect.

**Methodology/Principal Findings:**

Human ASCs were harvested from female lipoaspirate. Critical-sized (4 mm) calvarial defects were created in the parietal bone of adult male nude mice. Defects were either left empty, treated with an apatite coated PLGA scaffold alone, or a scaffold with human ASCs. MicroCT scans were obtained at stratified time points post-injury. Histology, *in situ* hybridization, and histomorphometry were performed. Near complete healing was observed among hASC engrafted calvarial defects. This was in comparison to control groups that showed little healing (*P<0.01). Human ASCs once engrafted differentiate down an osteogenic lineage, determined by qRT-PCR and histological co-expression assays using GFP labeled cells. ASCs were shown to persist within a defect site for two weeks (shown by sex chromosome analysis and quantified using Luciferase+ ASCs). Finally, rBMP-2 was observed to increase hASC osteogenesis *in vitro* and osseous healing *in vivo*.

**Conclusions/Significance:**

Human ASCs ossify critical sized mouse calvarial defects without the need for pre-differentiation. Recombinant differentiation factors such as BMP-2 may be used to supplement hASC mediated repair. Interestingly, ASC presence gradually dissipates from the calvarial defect site. This study supports the potential translation for ASC use in the treatment of human skeletal defects.

## Introduction

Current surgical strategies for the healing of skeletal tissue defects employ either autogenous grafts or alloplastic materials. Although current approaches are in large part successful, they come with inherent disadvantages. For example, alloplastic materials have problems with rejection, infection and eventual breakdown. Autologous tissues such as bone and bone marrow grafts are often limited in availability, and require a substantive operation with potential morbidity [1], [2]. Thus, there remains a pressing need for a suitable alternative to currently available techniques for bone tissue repair. Our laboratory and others have focused on harnessing the osteogenic capability of adipose-derived stromal cells (ASCs) for the eventual repair of non-healing skeletal defects [3]–[7].

Human adipose derived stromal cells (hASCs) are isolated from the stromal vascular fraction of human lipoaspirate. They have been described as a mesodermal stromal cell, with a proven ability to differentiate along osteogenic, adipogenic, chondrogenic and myogenic cell types, among others [8]–[19]. ASCs offer several advantages over other multipotent cells (such as bone marrow mesenchymal cells) for tissue engineering purposes [6], [18], [20]. ASCs are easily obtainable by a commonly used surgical procedure. ASCs are highly proliferative and thus are available in abundance. Moreover, hASCs show robust mineralization within one week of *in vitro* differentiation [21]. Previous studies have attempted to utilize hASCs for the regeneration of skeletal defects, but have met with limited success [22], [23]. Our study sought to assess the capacity of freshly derived and undifferentiated human ASCs to regenerate a non-healing mouse defect.

Our laboratory and others have previously employed a calvarial defect model for both the evaluation of normal healing, and the use of ASCs for the healing of critical sized (or non-healing) defects [24]–[30]. Previously, we have demonstrated that ASCs of mouse origin successfully heal a critical sized mouse defect [30]. In an effort to realize the bench to bedside application of ASCs in regenerative medicine, we now have examined the use of ASCs of human origin to heal calvarial defects. Human and mouse ASCs differ in multiple fundamental aspects [31], [32], and so this leap from mouse to man is by no means insignificant. To avoid incompatibility of xenografted tissue, an athymic mouse model was utilized (Charles Rivers, Crl:CD-1 *Foxn1^nu^*) [33]. Herein, we observed that freshly isolated human ASCs successfully heal a critical sized mouse calvarial defect. Subsequently we demonstrate that we can augment this osteogenic healing by supplementing BMP-2.

## Results

### Human ASCs undergo *in vitro* osteogenic differentiation and can be successfully grafted unto a calvarial defect

**Figure 1:**
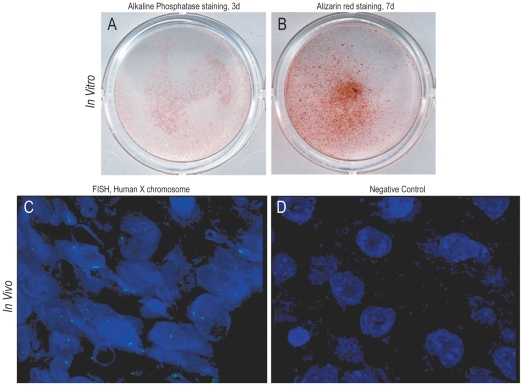
Human ASC Differentiation and Engraftment. (A,B) Human ASCs undergo *in vitro* osteogenic differentiation. (A) Gross photograph of alkaline phosphatase staining at 3 days differentiation. (B) Gross photograph of alizarin red staining at 7 days differentiation. (C,D) Fluorescent *in situ* hybridization for human X chromosome, appearing green. Nuclear counterstain appearing blue. (C) As expected, the majority of cells within the defect site at one week were of human origin, showing two X chromosomes. (D) Specificity of FISH analysis was ensured, as sites other than the defect were negative.

First, the osteogenic differentiation of human (h)ASCs was verified using standard osteogenic differentiation medium (ODM) *in vitro* over a period of seven days (**Figure 1A,B**). Alkaline phosphatase enzymatic activity was assessed at 3 days differentiation, which appears purple and is representative of early osteogenic differentiation (**Figure 1A**). Bone nodule formation was visualized after 7 days differentiation, as assessed by Alizarin red S staining (**Figure 1B**). In both cases, hASCs showed robust staining indicative of *in vitro* osteogenic differentiation.

Next, successful hASC *in vivo* cell engraftment was confirmed. PLGA scaffolds were seeded as described in the methods section. Representative animals from each group were sacrificed at 1 week. Cells were stained with DAPI nuclear counterstain, appearing blue. Fluorescent *in situ* hybridization (FISH) was performed specific for human sex chromosomes. This was performed to confirm viability of hASCs directly engrafted, as well as their cell progeny. Results showed, as expected, that those cells within the defect site were positive for human-X chromosome (**Figure 1C**). In contrast, those cells not within the defect site were negative, confirming the success of our xenograft and the specifity of our FISH analysis (**Figure 1D**). Thus, not only were hASCs successfully engrafted, they remained viable and contained within the defect site. Having demonstrated successful hASC engraftment, we next inquired as to whether hASCs would successfully heal this surgically created defect.

### Human ASCs heal critical size mouse calvarial defects by gross examination

**Figure 2:**
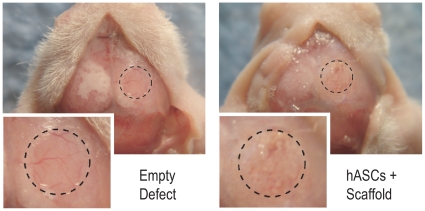
Gross appearance of calvarial defects. (A,B) Four millimeter calvarial defects were created in the right parietal bone of male p60 nude mice. After 8 wks healing, mice were sacrificed, a longitudinal incision was made over the cranium, and the skull was exposed for photographic documentation. On the left, a representive empty defect, in which very little osseous healing as occurred. Thus, the brain, meninges and meningeal vessels are visualized. On the right, a representative defect grafted with hASCs on a PLGA scaffold. Neither brain nor meninges are visible. Instead a new layer of woven bone has healed the critical size defect. Dashed lines encircle the original defect. N = 5 per group in total. Box in bottom left is a magnified view.

First, three experimental groups were assayed over 8 weeks healing post injury: 1) empty defects, 2) scaffold only, and 3) undifferentiated hASCs in combination with a scaffold (*n* = 5 per group). After 8 weeks, all mice were sacrificed for gross and histological analysis. Bird's eye photographs after 8 weeks verified a near complete lack of healing in empty defects (**Figure 2**). Thus the brain, meninges and meningeal vessels were grossly apparent (left, **Figure 2**). In marked contrast, those defects engrafted with hASCs showed robust bony regenerate throughout (right, **Figure 2**).

### Human ASCs heal critical size mouse calvarial defects by microCT

**Figure 3:**
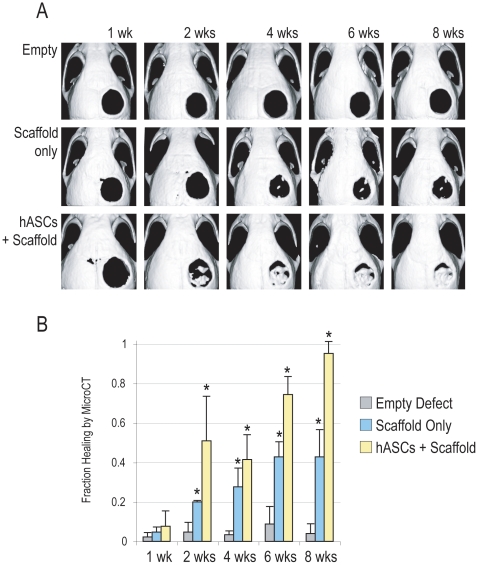
Calvarial healing by microcomputed tomography. Four millimeter calvarial defects were created in the parietal bone of p60 nude mice. Treatment groups included empty defects, defects treated with scaffold only, or bone defects treated with hASCs within a scaffold. (A) MicroCT scanning up to 8 weeks revealed near complete lack of healing among empty defects. Small peninsulas or islands of bone nodule formation were observed among scaffold only defects. In marked contrast, hASCs impregnated scaffolds were observed to nearly heal within 4 weeks. (B) Quantification of MicroCT. At up to 8 wks, healing of defects represented as a fraction of total defect area was quantified by microCT images. N = 5 per group, *P<0.01.

To assess bone regeneration, microCT scans were next performed at time 0 as well as 1, 2, 4, 6 and 8 wks post injury. As expected of a critical sized defect, those defects which were left empty showed little or no healing by microCT at up to 8 wks (top row, **Figure 3A**). In comparison, defects treated with scaffold only showed small peninsulas or islands of bony regenerate as well as some healing from defect edges inward, but the majority of the defect showed no osseous healing (middle row, **Figure 3A**). Finally, defects treated with hASCs grafted onto a scaffold showed robust healing (bottom row, **Figure 3A**). By only 4 weeks post-injury, near complete healing was appreciated by microCT. Thus, by both gross examination and microCT imaging, it appeared that hASCs when grafted onto a PLGA scaffold successfully heal a critical size mouse calvarial defect.

Quantification of microCT images was next performed. Percentage healing of the defects was evaluated by quantifying pixels in the defect using Adobe PhotoShop. Percentage healing was determined by dividing the defect area by the defect size immediately postoperatively (**Figure 3B**). Results showed that empty defects healed by less than 10% over the course of 8 wks (grey bars, **Figure 3B**). Defects treated with scaffold only showed a significant increase in healing percent, amounting to 42% after 8 wks (blue bars, **Figure 3B**). Finally, defects treated with hASCs showed an approximate 75% healing after 6 wks and completely healed by 8 weeks (yellow bars, **Figure 3B**).

### Human ASCs heal critical size mouse calvarial defects by histological analysis

**Figure 4:**
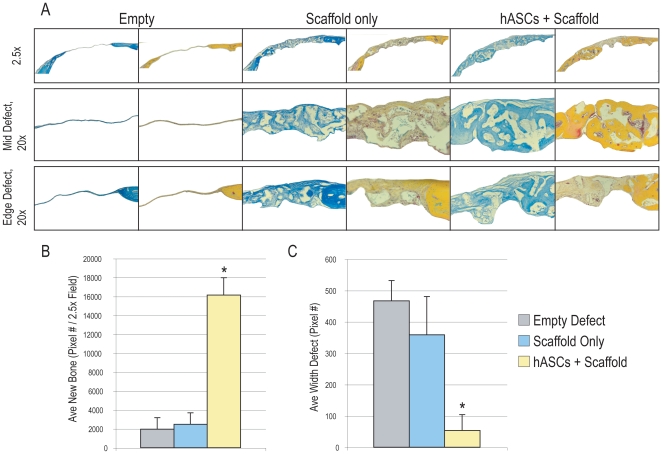
Calvarial healing by histology. (A) Histology at 8 weeks post injury. Serial sections throughout the defect site were created at 8 weeks post injury. Representative slides were stained with aniline blue, in which osteoid appears dark blue. As well, select slides were stained with Pentachrome, in which bone appears bright yellow. At low magnification (2.5x) the empty defects showed complete lack of new bone formation (left columns). Some small amount of woven bone was appreciated among defects treated with an empty scaffold (middle columns). However, near complete ossification was observed in defects treated with hASCs in a scaffold (right columns). This was accompanied by an increased thickness in the bony regenerate. (B) Quantification of bone formation at 8 weeks post injury by histological analysis. Average bone formation from every tenth slide was obtained using Adobe Photoshop analysis of aniline blue staining. Results showed a significant increase in bone formation with hASCs in comparison to other groups. (C) Average defect width was calculated on the same specimens. As expected a significant decrease in defect width was appreciated with hASC implantation, with many slides showing complete healing. N = 5 per group, *P<0.01.

We next sought to verify our gross and microCT findings by direct histological examination. Serial sections were generated through each defect; approximately 100 slides were made through each defect. Every tenth slide was stained to examine the entirety of the defect (**Figure 4A**). Results showed that empty defects had little or no bony regenerate by 8 wks by both aniline blue and pentachrome staining (top left, **Figure 4A**). The mid defect showed only dural tissue, without evidence of bone (middle left, **Figure 4A**). Finally, the defect edge showed an abrupt cutoff from old bone to absence of bone (bottom left, **Figure 4A**). In effect, no ossification was observed among empty defects. Among scaffold only defects, some ossification was apparent by aniline blue and pentachrome (middle columns, **Figure 4A**). This was most apparent at the defect edge, where a thin layer of osteoid was apparent on the ectocranial surface (middle columns, **Figure 4A**). Finally, and in marked contrast to the other groups, robust trabeculated bone formation was apparent in hASC treated defects (right columns, **Figure 4A**). For example, large amounts of yellow stained woven bone were appreciated throughout the defect site (right columns, **Figure 4A**).

Next, by histomorphometric measurements we sought to substantiate our findings (**Figure 4B,C**). New bone regenerate within the defect site was measured using Adobe Photoshop as previously described [34]. As well, defect width was measured on the same slides. Results showed a significant increase in new bone area among hASC treated defects (**Figure 4B**). This was accompanied by a substantial decrease in the defect width (**Figure 4C**). Thus, by gross analysis, microCT imaging and histological examination, human ASCs were observed to heal a critical size mouse calvarial defect.

### ASCs directly undergo osteogenic differentiation *in vivo*

**Figure 5:**
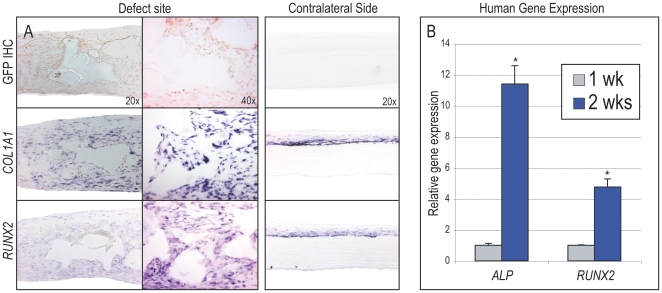
Human ASCs undergo osteogenic differentiation *in vivo*. Human ASCs transduced with a GFP encoding lentivirus were seeded in calvarial defects. (A) Histological analysis at 2 wks postoperative. The defect site (left) and contralateral aspect of the skull (right) from identical sections are imaged to show specificity of staining. (A, top) GFP immunohistochemistry, depicting evidence of human cells which appear brown. The contralateral side shows no GFP signal demonstrating the specificity of the stain. (A, middle) *Type I Collagen (COL1A1)* expression by *in situ* hybridization. (A, bottom) *Runt related transcription factor 2 (RUNX2)* expression by *in situ* hybridization. Staining can be seen in the uninjured contralateral periostium demonstrating specificity and can also be seen within the defect site. (B) Human osteogenic gene expression by qRT-PCR at 1 and 2 wks post-injury among hASC engrafted defects, including *hALP*, *hRUNX2*, *P<0.01.

ASC engraftment led to defect ossification by all markers examined, but do hASCs directly undergo *in vivo* osteogenic differentiation? To answer this hASCs transduced with a human lentivirus encoding green fluorescent protein (GFP), permitting *in situ* detection of human cells were utilized. Next, GFP immunohistochemistry was performed on GFP+ hASC engrafted defects at 2 week postoperatively. At 2 wks, GFP+ cells were detected in the defect site (**Figure 5A** **, upper left**). As a negative control, the contralateral (or uninjured) side of the calvaria was imaged, which verified the specificity of GFP immunostaining **(** **Figure 5A** **, upper right**). Next, staining for osteogenic markers (*COL1A1* and *RUNX2*) was performed on adjacent slides to those used for GFP analysis, using *in situ* hybridization (**Figure 5A** **, middle and bottom rows**). Results showed that those cells which were GFP+ (indicating human origin) also stained for osteogenic markers. We also show the contralateral uninjured side of the defect to demonstrate the specificity of the stain which can be seen in the periostium (**Fig. 5A** **, right column**).

As further verification, qRT-PCR analysis was performed on calvarial defects for the presence of human osteogenic genes (**Figure 5B**). Expression of human *(h)GAPDH, hALP* and *hRUNX2* expression were found at both 1 and 2 wks. As a negative control, defects without hASC engraftment showed no amplification, confirming specificity for human genes (data not shown). Moreover, both *hALP* and *hRUNX2* showed increased expression from 1 to 2 wks when normalized to *hGAPDH*. Thus and in summary, these data suggested that hASCs express osteogenic genes *in vivo* during a time period corresponding to cranial defect ossification, a finding supporting osteogenic differentiation of engrafted hASCs.

### ASCs do not persist in the healed mouse calvarial defect beyond two weeks

**Figure 6:**
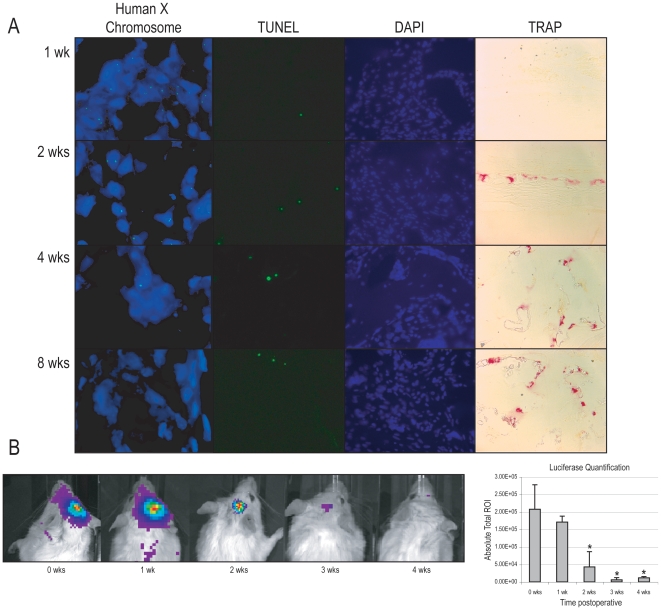
Bone turnover within a calvarial defect. (A, far left column) FISH staining for human sex chromosomes in hASC engrafted defects from 1–8 weeks postoperatively. Human X chromosome appears green, while DAPI nuclear counterstain appears blue. 100× magnification. (A, mid left column) TUNEL staining in hASC engrafted defects from 1–8 weeks postoperatively. TUNEL positive cells appear green, while DAPI nuclear counterstain appears blue. (A, far right column) TRAP staining in hASC engrafted defects from 1–8 weeks postoperatively. TRAP positive cells appear red/purple. (B) Luciferase activity in defects engrafted with Luc+ mouse ASCs. Images from a representative mouse (left), and quantification (right). Red represents the highest level of activity, followed by yellow, blue, dark blue, and purple. N = 3 animals. *P<0.01.

Our prior FISH analysis proved successful initial hASC engraftment and osteogenic differentiation, but what happens to engrafted hASCs overtime? For this purpose, we again employed FISH analysis for human sex chromosome at 1, 2, 4 and 8 weeks postoperatively (**Figure 6A**). At 1 week post-injury, the majority of cells within the defect site stained positively for human X chromosome, indicating their hASC origin (**Figure 6A**, left column). At 2 weeks, numerous individual cells still stained positively, however they were interspersed with areas in which cells were distinctly of mouse origin (**Figure 6A**, left column). At 4 weeks and 8 weeks, a virtual absence of FISH positive cells was identified (**Figure 6A**, left column). This somewhat surprising result was verified by qRT-PCR at 8 weeks. 1 ug of RNA from the defect site was reverse transcribed. Using standard parameters commonly employed in our laboratory, human genes did not reach Ct values after 40 cycles. During the same assay, mouse *Gapdh* showed robust amplification (data not shown). Taken together, these data suggest that although hASC engrafted defects successfully heal, human cells do not persist in the defect site. Two possibilities for this were further investigated. First, hASCs could not persist due to cell death. TUNEL assays were performed at 1, 2, 4 and 8 weeks. Sparse TUNEL + cells were noted within the defect site at all time points (**Fig. 6A**, middle column), thus cell death could not explain the apparent lack of human cell persistence within the defect site. Second, active bone turnover could explain this phenomenon, such that hASC produced bone may have been gradually replaced overtime with mouse host derived tissue. Interestingly, this did appear to be the case, as an increasing intensity of TRAP staining was noted in the defect site from 2–8 weeks postoperatively (**Fig. 6A**, right column). Taken together, these data suggest that hASC-engrafted PLGA scaffolds heal a critical size mouse calvarial defect, however, by 8 weeks the healed defect is predominantly of host mouse origin.

In our previous study utilizing mouse ASCs in a calvarial defect, we observed the persistence of mouse donors cells at 2 weeks postoperatively [30]. In more detail, we sought to ascertain whether mouse ASCs in similarity to hASCs did not persist overtime. For this purpose, mASCs were harvested from transgenic mice expressing Firefly Luciferase (**Fig. 6B**). Luc+ mASCs were engrafted in wild-type CD-1 mice and Luciferase activity was imaged weekly thereafter (**Fig. 6B**). At 0 through 2 weeks, luciferase activity was observed in the defect site, however the quantity of luciferase activity was noted to significantly attenuate thereafter (right, **Fig. 6B** for quantification). Thus in summary, among ASCs of both mouse and human origin, a significant dissipation in cell number was observed in the initial two weeks following implantation.

### BMP-2 enhances human ASC mediated bony healing *in vivo*

**Figure 7:**
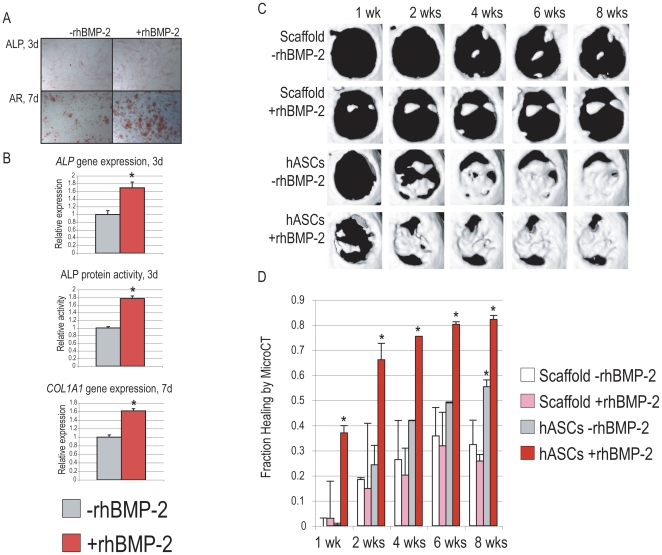
BMP-2 increases hASC osteogenesis *in vitro* and *in vivo*. (A,B) Recombinant human (rh)BMP-2 was first added to standard osteogenic differentiation medium (ODM) to verify its effects on in vitro hASC osteogenic differentiation. (A, top) Alkaline phosphatase (ALP) staining, appearing purple, at 3 days differentiation with or without rhBMP-2 (100 ng/ml). (A, bottom) Alizarin red staining, appearing red, at 7 days differentation with or without rhBMP-2 (100 ng/ml). (B) From top to bottom: relative ALP gene expression by qRT-PCR at 3 days, relative ALP enzymatic activity normalized to protein content at 3 days, and relative COL1A1 gene expression by qRT-PCR at 7 days. (C,D) Next, rhBMP-2 was delivered to hASC-engrafted defects by subcutaneous injection on days 1–3 postoperatively. Healing was assessed by serial microCT examination and quantification (C) MicroCT healing from 1–8 weeks with hASCs alone or hASCs + rhBMP-2. (D) Quantification of fraction healing from 1–8 weeks postoperatively. N = 3, *p<0.05.

Various differentiation factors have been shown to enhance *in vitro* hASC osteogenic differentiation, including bone morphogenetic protein (BMP)-2 [29], [32]. We first sought to verify the pro-osteogenic effect of rhBMP-2 on hASC *in vitro* differentiation [35]. Osteogenic differentiation was performed over 1 week in culture with or without rhBMP-2 (100 ng/ml, a concentration derived from dose curves performed). By all markers examined, osteogenic differentiation was significantly enhanced by rhBMP-2 addition to ODM, (**Fig. 7A,B**). This included alkaline phosphatase staining and quantification, Alizarin red staining of bone nodules, and specific gene expression by qRT-PCR (*ALP*, *COL1A1*). We next sought to substantiate the use of rhBMP-2 *in vivo*: a cytokine suspension was delivered to the defect site via simple subcutaneous injection on postoperative days 1–3 and followed by microCT (**Figure 7 C,D**). Results showed that significant healing was observed among control hASC engrafted defects, over 55% after 8 weeks healing (**Fig. 7C**, top). In comparison, significantly more robust healing was observed with addition of rhBMP-2 to hASC treated defects (**Fig. 7C**, bottom). This was subsequently quantified, yielding a significant increase in bone formation among rhBMP-2/hASC treated defects (**Fig. 7D**).

## Discussion

This study extends what is already known regarding the potential use of ASCs for osseous tissue repair and regeneration. Prior studies have focused on ASCs from multiple species, including mouse, rabbit and human [9], [18], [22]. ASCs have a capacity to undergo rapid osteogeneic differentiation, and in our observations show robust mineralization within one week *in vitro* [36]. Our laboratory and others have documented the ability of ASCs derived from rodent species to heal surgically created cranial defects [22], [23], [30]. Our study sought to address two specific questions. First, can ASCs of human origin regenerate a non-healing calvarial defect? Secondly, can this be performed without the need for pre-differentiation? We believe this second question to be clinically relevant, as the less time hASCs need to expand in *vitr*o, the less likelihood of *in vitro* contamination, the timelier the transfer to *in vivo* host, and perhaps most importantly the shorter the in-hospital patient stay.

We observed that freshly isolated, undifferentiated hASCs successfully healed a critical sized cranial defect. Interestingly, we observed a much more rapid healing of defects engrafted with human ASCs than that of previous studies in ASCs of other species [30]. The fact that substantive differences exist between mouse and human ASCs upon *in vivo* engraftment is in part to be expected, as vast *in vitro* differences do exist. For example, human ASCs undergo much more rapid *in vitro* osteogenesis [32]. Moreover, hASC osteogenesis goes unperturbed even in the presence of growth factors such as FGF-2 or TGF-β1 [31]. These differences are exciting for multiple reasons. First, if we are able to fully document those factors that play a role in the enhanced osteogenesis observed in human as compared to mouse cells, we may further capitalize on them via gene or protein manipulation. Secondly, the robust osteogenic response of human ASCs suggests that in the future even a relatively large sized defect may be successfully repaired via hASC autologous transfer.

In fact, small studies have already examined the potential use of hASCs to heal skeletal defects in the human patient. Defects of the cranium [37], maxilla and mandible [38] have been either healed or enabled to heal faster with the use of hASCs [39]. Methods of hASC usage have varied dramatically and have included combination with bone chips, the use of various osteoconductive scaffolds as well as recombinant proteins. For example, Mesimaki *et al.* used a novel microvascular flap with hASCs, beta-tricalcium phosphate and BMP-2 to heal a large defect in the maxilla, reporting good outcomes up to 8 months post-operatively [40]. Larger scale studies must verify these findings, however we believe that the optimum delivery method and cytokine stimuli can be fine tuned utilizing our nude mouse model.

Our study also found that hASCs require no special additions to medium prior to engraftment. Various cytokines and other factors are known to stimulate osteogenic differentiation among ASCs. These include bone morphogenetic proteins (BMPs) [32], Insulin like growth factor-1 (IGF-1) [21], histone deacetylase inhibitors (HDIs) such as valproic acid [34], and Sonic Hedgehog [36]. We found that the use of such factors (in this study BMP-2) in addition to hASC engraftment more rapidly healed a defect. BMPs have been thoroughly studied in the differentiation of ASCs by methodologies including addition of recombinant protein, addition of neutralizing antibodies, viral over expression and siRNA [22], [32], [41], [42]. These studies suggest that BMP signaling is sufficient to enhance ASC osteogenic differentiation and conversely BMP/BMPR1B signaling necessary for the normal osteodifferentiation to occur. Various methods may exist for the potential delivery of such growth factors. Simple subcutaneous injection was sufficient in the present study. However, a more sophisticated approach may utilize hASCs engrafted onto a defect laden or impregnated with growth factors to promote even more robust bone formation. Alternatively, a short *in vitro* pretreatment of hASCs followed by *in vivo* engraftment without recombinant protein may be just as advantageous without potentially stimulating neoosteoclastogenesis [27], [29]. The benefit of the technique presented in this manuscript, however, is that it does not require any pre-treatment of the hASCs. This ability to use the harvested hASCs immediately will allow the surgeon to perform a reconstruction in one step and also may be advantageous from a Food and Drug Administration (FDA) standpoint. Thus we anticipate the use of our nude mouse model for the future fine-tuning of hASC mediated calvarial regeneration.

One potentially puzzling aspect of our study was the fact that hASCs were not found to predominantly persist within our cranial defect model after 2 weeks engraftment. Prior studies have examined the long-term *in vivo* survival of human ASCs in an immunocompromised mouse similar to our study. For example, after intravenous injection, hASCs were found to persist within the mouse even after 8 months [43]. In contrast, the simple subcutaneous injection of hASCs was found to dissipate after as little as 5 days in other models [44]. In current studies in our laboratory, fat grafting into a subcutaneous pocket has been performed using human tissue in the same athymic CD-1 mouse. We have thus far shown continued presence of human cells up to 3 months postoperatively (data not shown). A bone defect is a much different *in vivo* environment, however, which has both inflammation and bone turnover. How would these factors influence engrafted hASC persistence? Our results suggested that the persistence of hASCs within a calvarial defect site is limited to approximately two weeks. This was not explained by impaired cell survival, but rather by clear bone turnover within the defect site. Interestingly, these findings were recapitulated by the use of mouse ASCs expressing Firefly Luciferase, suggesting that this finding is not species specific. We would argue that these findings enhance rather than diminish the significance of our results. An ideal substance for reconstruction, whether prosthetic or autologous, would be one that stimulates the host's reparative processes so that the final healed tissue is of host origin. Thus, the armamentarium of a future reconstructive surgeon could consist for example of biomimetic, biodegradable scaffolds through which host tissue could easily migrate. It appears from our study that ASCs complement this paradigm nicely, as their engraftment led to complete healing a calvarial defect, but without long-term persistence of the foreign xenografted material. Thus, while dissipation of fat grafts are of disadvantage in soft tissue augmentation for example, this same phenomenon may be of real benefit in the case of cell-based strategies for skeletal reconstruction.

In conclusion, undifferentiated human ASCs successfully heal critical sized mouse calvarial defects. Thus, human ASCs represent a promising cell type for future translational efforts in the autogenous repair of skeletal defects. BMP signaling may beneficially modulate hASC mediated bony repair. We believe that the hASC/nude mouse model has utility in the *in vivo* optimization of hASC mediated skeletal tissue regeneration.

## Methods

### Ethics Statement and Human ASC Harvest

ASCs were harvested from human lipoaspirate as previously described [21]. All research involving hASCs has been approved by the Stanford institutional review board, protocol #2188 and #9999. Written informed consent was obtained for all patients, information regarding patient age and sex was recorded, in all other respects anonymity was maintained (**Figure S2, S3**). Briefly, specimens were washed in dilute Betadine, then phosphate buffered saline (PBS), and digested with a 0.075% Type II collagenase in Hank's Balanced Salt Solution at 37 degrees Celsius under agitation for 30 min. Next, collagenase was inactivated by 10% Fetal Bovine Serum (FBS) in PBS. The stromal vascular fraction (SVF) was then pelleted, the supernatant discarded, and the cell pellet resuspended and filtered through a 100 micrometer strainer. Primary cultures were established at 37 degrees Celsius, 21% O_2_, 5% CO_2_. For all assays, specimens were obtained from female patients under fifty years of age, from the flank and thigh subcutaneous regions only. In total, hASCs were pooled from five patients for all assays. Mean patient age was 46.5 years, mean BMI was 26.1 kg/m^2^, with all patients weighing less than 30 kg/m^2^. In addition, for select experiments mouse ASCs were derived from the inguinal fat pads [34] of transgenic CD-1 mice with constitutively active Firefly luciferase in order to quantify cell persistence upon *in vivo* engraftment. Cells were expanded for 72 hours in growth medium (DMEM, 10% FBS, 1% penicillin/streptomycin), passaged by trypsinization and placed on scaffolds or seeded for subsequent *in vitro* experiments.

### *In vitro* differentiation assays

#### To verify that hASCs were capable of osteogenic differentiation and determine their responsiveness to recombinant human (rh)BMP-2, *in vitro* assays were performed

Human ASCs were plated in 6 well plates at a concentration of 100,000 cells/well. Cells were treated with standard osteogenic differentiation medium (ODM) with or without recombinant human (rh)BMP-2 (100 ng/mL). Dose curves were performed to arrive at this optimum concentration (10–400 ng/mL). Alkaline phosphatase staining and quantification of enzymatic activity was performed at 3 days differentiation as previously described [21]. Alizarin red staining was performed at 7 days differentiation as previously described [45]. Specific gene expression was analyzed at 3 days differentiation by quantitative RT-PCR, standardized to housekeeping gene expression [46]. Quantitative real-time polymerase chain reaction was carried out using the Applied Biosystems Prism 7900HT Sequence Detection System. Specific primers are listed in **Figure S1**.

### GFP Transfection

First passage hASCs were allowed to reach 80% confluency in a 10 cm plate. Subsequently, cells were treated with a turboGFP lentiviral construct (Open Biosystems) in regular growth media in the presence of 8 ug/ml plybrene. After 12 hours, the cells were observed to have green fluorescence verifying transfection. Cells were subsequently treated with puromycin to select only those cells successfully transfected.

### Calvarial Defects

Animals were obtained from Charles Rivers laboratories and housed in the Research Animal Facility on Stanford University campus. The facility, accessed by authorized personnel only, is temperature, ventilation and illumination controlled. Mice have access to feed and water *ad libitum*. All mice housing conforms to NIH *Guide* standards, the Animal Welfare Act, and ILAR *guide*. Transportation of animals was performed based on the “Guidelines for Transportation of Animals from the Stanford Centralized Animal Facilities,” developed by the Administrative Panel on Laboratory Animal Care (A-PLAC). All animal procedures were approved by Stanford A-PLAC, Protocol #9999. Non-healing, critical-sized (4 mm) calvarial defects were created in the right parietal bone of adult (60 day-old) male CD-1 nude mice (Charles Rivers, Crl:CD-1 *Foxn1^nu^*) using a high-speed dental drill. For mouse ASC experiments, CD-1 wildtype mice were used. Animals were anesthetized using 20 mg/kg Ketaset, 1.5 mg/kg xylazine, and 0.2 mg/kg acepromazine maleate. After cleaning the surgical site with Betadine, an incision was made just off the sagittal midline to expose the right parietal bone. The pericranium was removed using a sterile cotton swab. Using diamond-coated trephine bits and saline irrigation, unilateral full-thickness critical-size calvarial defects were created in the non-suture associated right parietal bone. Importantly, the dura mater was left undisturbed.

In preparation for implantation, scaffolds were seeded with female hASCs. 150,000 cells were resuspended in 25 µl of growth media (DMEM, 10% FBS, 1% penicillin/streptomycin), and placed directly onto the scaffold for 30 minutes (25 µl of media without cells were used for empty scaffold controls). The scaffolds were subsequently submerged in 100 µl of growth media for 24 hours incubation. Before implantation, cell-seeded scaffolds were copiously rinsed in sterile PBS to prevent transfer of medium-derived growth factors or immunogens. Animals were split equally into three treatment groups: 1) empty defects in which a 4 mm defect was created but left empty, 2) scaffold only, in which a PLGA scaffold without cells was placed in the defect site and, 3) hASCs and scaffold, in which hASCs were impregnated in a scaffold (*n* = 5 per group). Finally, the skin was sutured with 6-0 vicryl and the animal was monitored per established post-operative animal care protocols. Animals were allowed to recover under heat lamps for a period of up to 6 hours before being returned to the animal facility. Thereafter, animals were observed once daily for three days and weekly thereafter to ensure postoperative recovery.

In order to more robustly heal defects, recombinant human (rh)BMP-2 was delivered via subcutaneous injections. Subcutaneous injections of a 50 ul cytokine suspension in normal saline were performed on days 1, 2 and 3 postoperatively (500 ng BMP-2 daily in 50 ul) (R&D systems). Animals were sedated with Aerrane and a wheal was made just overlying the defect site. Animals were allowed to recover before being returned to the animal facility. Four groups were performed: 1) scaffold only with vehicle injection as a control, 2) scaffold only with rhBMP-2 injection, 3) hASC engrafted scaffold with vehicle injection as a control, and 4) hASC engrafted scaffold with rhBMP-2 injection (N = 3 per group).

Finally, for select experiments we utilized transgenic Luciferase+ CD-1 mice. Luc+ mASCs, derived from inguinal fat pads were engrafted per the above protocol into parietal defects in wild-type CD-1 adult (60 day old) mice (N = 3).

### Scaffold creation

Apatite-coated PLGA scaffolds were fabricated from 85/15 poly(lactic-co-glycolic acid) (inherent viscosity  = 0.61 dL/g, Birmingham Polymers) by solvent casting and a particulate leaching process. Briefly, PLGA/chloroform solutions were mixed with 200−300 m diameter sucrose to obtain 92% porosity (volume fraction), and compressed into thin sheets in a Teflon mold. After freeze-drying overnight, scaffolds were immersed in three changes of double-distilled (dd) H_2_O to dissolve the sucrose, and gently removed from the Teflon plate with a fine-tip spatula. After particulate leaching, all scaffolds were disinfected by immersion in 50%, 60% and 70% ethanol for 30 min each, followed by three rinses of ddH_2_O. All scaffolds are then dried under a laminar flow hood.

### *In Vivo* Imaging

Micro computed tomography was performed, using a high-resolution MicroCAT II™ (ImTek Inc., Knoxville, TN) small animal imaging system. Live animals were imaged after sedation with Aerrane (Isoflurane, Baxter Healthcare Corporation, Deerfield IL). The following settings were used: x-ray voltage of 80 kVp, anode current of 500 µA and an exposure time of 500 milliseconds for each of the 360 rotational steps. The 2D projection images were used to reconstruct tomograms with a Feldkamp algorithm, using a commercial software package (Cobra EXXIM, EXXIM Computing Corp., Livermore, CA), resulting into a resolution of 80 µm. The duration of one scan was 9.5 minutes. 3D reconstructions were generated by MicroView software (GE Healthcare, London, Canada). Every mouse was scanned with a CT-phantom and isosurface was set in Hounsfield units according to the phantom which included hydroxyapetite, water and air.

In order to track Firefly Luciferase activity after engraftment of Luc+ mASCs, the *In Vivo* Imaging System (IVIS) was used. Mice were anesthetized and luciferin (150 mg/kg in 200 µL) injected into the peritoneal cavity. After 10 minutes, animals were then placed in the IVIS 200B™ imaging system and imaged for 3 minutes at large binning. Luciferase activity at the calvarial injury site was quantified weekly postoperatively using Living Image 3.2.

### Histologic Analyses

At 1, 2, 4 and 8 weeks, animals were sacrificed by CO_2_ asphyxiation and cervical dislocation to confirm radiographic findings. Calvaria were harvested, formalin-fixed, decalcified in 19% EDTA and paraffin-imbedded. Aniline blue staining was performed on every 10^th^ section throughout the sample to provide detailed histology of the regenerate. Histomorphometry was performed on every 10^th^ slide throughout the defect using Adobe Photoshop as previously described [34]. Next, select slides were stained with Pentachrome, in which bone appears bright yellow. Alkaline phosphatase staining was performed on select slides, as previously described [34], [47]. To examine cell death and bone turnover, TUNEL and TRAP staining was performed as previously described [47]. GFP immunohistochemistry was performed on select slides as previously described [36], per manufacturers instructions. Finally, *Runx2* and *Col1a1 in situ* hybridization was performed on select slides, as previously described [47].

### Fish Analysis for sex chromosomes

Tissue sections were pretreated by standard protocol using the VP2000™ slide pretreatment instrument (Abbott Molecular). Briefly, slides were deparaffinized, digested with a 10% pepsin solution at 37°C (Protease I, VP2000™ Protease Buffer; Abbott Molecular), pre-treated with a sodium thiocyanate solution at 80°C (VP2000™ Pretreatment Solution; Abbott Molecular), re-fixed in 10% buffered formalin, and dehydrated in an ethanol series. Slides were denatured with a Vysis®HYBrite instrument at 73°C for six minutes and hybridized for 48 hours at 37°C with a dual-color DXZ1/DYZ3 probe set, specific for the centromeres of the human X and Y sex chromosomes, respectively (Abbot Molecular). Slides were washed with 2xSSC/0.3% NP-40 at 73°C for two minutes, counterstained with DAPI and analyzed with an Olympus BX51 microscope, appropriate fluorescent filters and a CytoVision® imaging system (Genetix, San Jose).

### Polymerase chain reaction

RNA was isolated from formalin fixed paraffin embedded slides using the RecoverAll RNA kit (Ambion, Cat #AM1975). The defect site of 6 slides (approximately 24 sections) was used as tissue starting material. After quantification by spectrophotometry, reverse transcription was performed with Taqman Reverse Transcription Reagents (Applied Biosystems, Foster City, CA). Quantitative real-time polymerase chain reaction was carried out using the Applied Biosystems Prism 7900HT Sequence Detection System in triplicate wells [48]. Specific primers are listed in **Figure S1**.

### Statistical analysis

Means and standard deviations were calculated from numerical data, as presented in the text, figures and figure legends. In figures, bar graphs represent means, whereas error bars represent one standard deviation. Statistical analysis was performed using the ANOVA two-factor with replication when more than two groups were compared. In addition, the Welch's two-tailed *t*-test was used when standard deviations between groups were unequal. Inequality of standard deviations was verified by employing the Levene's test. *P≤0.01 was considered to be significant.

## Supporting Information

Figure S1 Sequences used in real-time polymerase chain reaction.
(0.03 MB DOC)

Figure S2 Informed Consent Sample. This document is a sample of our Informed Consent, which is signed by each human patient from which liposuction aspirate is harvested for cell derivation.
(0.06 MB DOC)

Figure S3 IRB Approval Letter. This IRB Approval Letter from the Stanford Institutional Review Board supports the harvest and study of human adipose derived stromal cells.
(0.09 MB PDF)
