# Supplementary material for: Human Adipose Derived Stromal Cells Heal Critical Size Mouse Calvarial Defects
Source: PLoS One. 2010 Jun 17;5(6):e11177. doi: 10.1371/journal.pone.0011177 (PMC2887361; doi:10.1371/journal.pone.0011177)
Supplement: Figure S1 — Sequences used in real-time polymerase chain reaction. (0.03 MB DOC) [file pone.0011177.s001.doc]

**SUPPLEMENTAL TABLE 1: PCR Primer Sequences**

| *Gene Name* | *Forward primer sequence (5’ to 3’)* | *Reverse primer sequence (5’ to 3’)* |
| --- | --- | --- |
| *ALP, human* | ATGGGATGGGTGTCTCCACA | CCACGAAGGGGAACTTGTC |
| *COL1A1, human* | ATGACTATGAGTATGGGGAAGCA | TGGGTCCCTCTGTTACACTTT |
| *GAPDH, human* | ATGGGGAAGGTGAAGGTCG | GGGGTCATTGATGGCAACAATA |
| *RUNX2, human* | ATTCCTGTAGATCCGAGCACC | GCTCACGTCGCTCATTTTGC |
