# Supplementary material for: Human Adipose Derived Stromal Cells Heal Critical Size Mouse Calvarial Defects
Source: PLoS One. 2010 Jun 17;5(6):e11177. doi: 10.1371/journal.pone.0011177 (PMC2887361; doi:10.1371/journal.pone.0011177)
Supplement: Figure S2 — Informed Consent Sample. This document is a sample of our Informed Consent, which is signed by each human patient from which liposuction aspirate is harvested for cell derivation. (0.06 MB DOC) [file pone.0011177.s002.doc]

| **Consent Form to Participate in Research** |
| --- |

*** * * * * * * * ***

**Informed Consent**

***Are you participating in any other research studies? ___yes ___no**

You are invited to participate in a research study of human fat and to help determine if human fat (adipose) tissue contains cells capable of growing into new and different kinds of tissue. You are being asked to donate your adipose tissue (human fat) which will be removed by your surgeon during your surgery. You were selected as a possible participant in this study because you have previously decided to undergo a surgical procedure which may provide otherwise discarded human adipose tissue at no additional burden or discomfort to you as a result of your willingness to participate. We hope to learn that human adipose has abundant precursor cells (cells that maintain the ability to form multiple tissue types) that when appropriately stimulated can develop into a multitude of tissues that may be used for regenerative therapies. There are several things you should know before allowing your tissue to be studied.

**Your participation in this study is entirely voluntary**. Your decision whether or not to participate will not prejudice you or your medical care. If you decide to participate, you are **free to withdraw** your consent and to discontinue participation at any time without prejudice to you or effect on your medical care.

If you decide to participate, we will obtain the tissue (adipose or fat) which was removed from you by your treating surgeon. This tissue is normally discarded and is considered medical waste. The tissue will be transported back to our laboratory at Stanford University. We will perform some chemical digests of this tissue in order to isolate the cellular elements which exist in all human adipose. We will then provide certain chemical stimulants to these cells in an effort to have them become a different type of cell such as a bone cell or heart muscle cell, rather than just a fat cell.

After isolation of the fat derived stem cells, we will also transfect some of these fat derived stem cells with different stem cell transcriptional factors. We hope to transform fat derived stem cells into induced pluripotent stem (iPS) cells. We hope these fat derived iPS cells can later be used for further medical research and test whether they can transform into other cells types such as neuronal, cardiac, hepatic, and endothelial cells, among others.

It is important that you understand the following regarding research on your donated tissue:

1. The derived cells or cell products may be kept for many years after you donate them.
2. No identification will be retained on the samples, so no one who works with them will know where they came from, so that even if we wanted to, we would not be able to contact you regarding specific cell samples.
3. Researchers may use cell lines for future studies, some of which may not be predictable at this time.
4. Derived cells or cell products may be used in research involving genetic manipulation.
5. Derived cells or cell products may be transplanted into humans or animals. Your decision to consent is being made without restriction on the recipient of any transplanted cells.
6. Derived cells or cell products are not intended to provide direct medical benefit to you.
7. Your decision to consent or refuse to donate materials for research will not affect the quality of any future care provided to you by Stanford.

Do you agree to donate your tissues for future research as described above (such as study of whether pluripotent stem cells can transform into other cells types such as neuronal, cardiac, hepatic, and endothelial cells)? ___Yes ___No

Please indicate if there are any restrictions on future uses of donated materials:

__ No restrictions __ Restrictions (Comment)___________________________________________

Researchers may choose to use materials only from donors who agree to all future uses without restrictions.

This is a one time commitment on your part if you choose to agree to allow us to study your adipose tissue. There are no risks or added discomfort to you as a result of your participation in this study. There are no additional tests or blood draws that need to be done as a result of your decision to participate. There are no reasonably foreseeable risks to you by your decision to participate.

There would be no direct benefit to you in agreeing to have your adipose tissue submitted for analysis in this study. Clinical medicine may benefit if we can identify potential pre-cursor cells present in this frequently abundant tissue reservoir (human adipose) that can robustly form bone. ***WE CANNOT AND DO NOT GUARANTEE OR PROMISE THAT YOU WILL RECEIVE ANY BENEFITS FROM THIS STUDY.**

You will be told if any new information is learned which may affect or influence your willingness to participate in this study. It is worth re-iterating that your commitment is only in submitting your adipose tissue for analysis on the day or your proposed surgery.

While participating in this study, you should not take part in any other research project without approval from all of the investigators. This is to protect you from possibly injury arising from such things as extra blood drawing, extra x-rays, interaction of research drugs, or similar hazards.

A decision to participate in this study is in no way related to your decision to have surgery. Your alternative is not to participate in this study. This decision will have no impact on your proposed surgery.

Your gender and age will be recorded with your tissue sample. Once the sample is taken, it will forever be separated, deidentified and unlinked from your name. This will protect your identity and preserve your anonymity. However, once you donate the sample, you will not be able to withdraw your tissues from the research project because the samples will not be traceable. Any useful information that may be gained from the study of your tissue may be published in scientific journals in which your identity could not be revealed since it will not be known by anyone. Patient information such as your gender and date of procedure would be the only relevant facts known about your tissue after it leaves your surgeon’s office.

No payment will be provided for participation in this project. There are no costs to you, your surgeon, or your insurance company as a result of your decision to participate in this study. Stanford University through the Department of Surgery is providing financial support to the investigators for this study. Any tissues you have donated which are used in research may result in new products, tests, or discoveries. In some instances, these may be patentable or have potential commercial value and may be developed and owned by the Investigators, Stanford University, and / or others. Under California law and rules, you as the donor will not receive patent rights and will not receive any financial or other benefit from future commercial development.

At the discretion of the investigators, subjects may be taken out of this study due to unanticipated circumstances. In addition, subjects may be taken out of the study without regard to your consent. Some possible reasons for withdrawing a subject from this study include:

-failure to follow instructions

-your tissue was not processed appropriately for purposes of study

-the study is canceled

-other administrative reasons

**Contact Information:**

- Questions, Concerns, or Complaints: If you have any questions, concerns or complaints about this **research study**, its procedures, risks and benefits, or alternative courses of treatment, you should ask the Protocol Director, (Dr. Michael T. Longaker at (650) 736-1704).  You should also contact him/her at any time if you feel you have been **hurt by being a part of this study**.
- *****Independent Contact: If you are not satisfied with how this study is being conducted, or if you have any concerns, complaints, or general questions about the research or your rights as a participant, please contact the Stanford Institutional Review Board (IRB) to speak to someone independent of the research team at (650)-723-5244 or toll free at 1-866-680-2906.  You can also write to the Stanford IRB, Stanford University, Stanford, CA 94305-5401.
- Appointment Contact (if applicable): If you need to change your appointment, please contact Dr. Michael T. Longaker (650 736-1704
- Alternate Contact (if applicable): If you cannot reach the Protocol Director, please call the research team at (650) 736-1704.

**Compensation**

All forms of medical diagnosis and treatment – whether routine or experimental – involve some risk of injury. In spite of all precautions, you might develop medical complications from participating in this study. If such complications arise, the Protocol Director and the researcher study staff will assist you in obtaining appropriate medical treatment. In the event that you have an injury or illness that is directly caused by your participation in this study, reimbursement for all related costs of care first will be sought from your insurer, managed care plan, or other benefits program. **You will be responsible for any associated co-payments or deductibles as required by your insurance.**

If costs of care related to such an injury are not covered by your insurer, managed care plan or other benefits program, you may be responsible for these costs. If you are unable to pay for such costs, the Protocol Director will assist you in applying for supplemental benefits and explain how to apply for patient financial assistance from the hospital.

Additionally, Stanford is not responsible for research and medical care by other institutions or personnel participating in this study. You do not waive any liability rights for personal injury by signing this form.

**Authorization to Use Your Health Information for Research Purposes**

Because information about you and your health is personal and private, it generally cannot be used in this research study without your written authorization. If you sign this form, it will provide that authorization. The form is intended to inform you about how your identifiable health information will be used or disclosed in the study. Your information will only be used in accordance with this authorization form and the informed consent form and as required or allowed by law. Please read it carefully before signing it.

**What is the purpose of this research study and how will my identifiable health information be utilized in the study?**

The purpose of this study is to investigate the potential to engineer new bone with cells derived from your discarded adipose tissue. In some cases, these cells may be affected by your overall health. Information regarding your health (e.g. diabetes, high blood pressure, age) will be recorded, but no personally identifying information except procedure date, along with gender and age, is transferred with the specimen; as such, the specimen is forever “unlinked” from your identity once it leaves the operating room. Your name will be retained on this signed consent/authorization form, which will be kept separately from study results under lock and key., which can only be accessed by the Protocol Director and authorized personnel.

**Do I have to sign this authorization form?**

You do not have to sign this authorization form. But if you do not, you will not be able to participate in this research study. Signing the form is not a condition for receiving any medical care outside the study. Your decision to sign or decline this form will not affect the medical care you receive before, during or after your surgery.

**If I sign, can I revoke it or withdraw from the research later?**

If you decide to participate, you are free to withdraw your authorization regarding the use and disclosure of your health information (and to discontinue any other participation in the study) at any time. After any revocation, your identifiable health information will no longer be used or disclosed in the study, except to the extent that the law allows us to continue using your information (e.g., necessary to maintain integrity of research). If you wish to revoke your authorization for the research use or disclosure of your health information in this study, you must contact:

Dr. Michael T. Longaker, MD, MBA, at (650) 736-1707

**What Personal Information Will Be Used or Disclosed?**

This consent/authorization form includes your name and your health condition. It will be kept separately from research results. The study will use certain other health information from you, but this will not be linked to your name or specimen, except by date of your procedure. This information that is not personally identifiable includes, but is not limited to, your age and your past medical history including any underlying conditions you may have such as diabetes, high blood pressure., which are alterations in your overall physiology that may affect potentially every cell in your body.

**Who May Use or Disclose the Information?**

The following parties are authorized to use and/or disclose your identifiable health information in connection with this research study:

- The Protocol Director: Dr. Michael T. Longaker, MD, MBA
- The Stanford University Administrative Panel on Human Subjects in Medical Research and any other unit of Stanford University as necessary
- Research Staff, or third parties hired by Stanford for legal or oversight purposes

**Who May Receive or Use the Information?**

The parties listed in the preceding paragraph may disclose your health information to the following persons and organizations if necessary for their use in connection with this research study:

- The Office for Human Research Protections in the U.S. Department of Health and Human Services
- The Food and Drug Administration (FDA)
- The National Institutes of Health (Bethesda, MD)

Your information may be re-disclosed by the recipients described above, if they are not required by law to protect the privacy of the information.

**When will my authorization expire?**

Your authorization for the use and/or disclosure of your health information will expire May 8, 2013. This date is arbitrary and will give us sufficient time to complete our studies.

________________________________

Signature of Participant

______________
Date

**Subject’s Bill of Rights**

As a human subject, you have the following rights. These rights include but are not limited to the subject’s right to:

- Be informed of the nature and purpose of the experiment
- Be given an explanation of the procedures to be followed in the medical experiment, and any drug or device to be utilized
- Be given a description of any attendant discomforts and risks reasonably to be expected
- Be given an explanation of any benefits to the subject reasonably to be expected, if applicable
- Be given a disclosure of any appropriate alternatives, drugs or devices that might be advantageous to the subject, their relative risks and benefits
- Be informed of the avenues of medical treatment, if any available to the subject after the experiment if complications should arise
- Be given an opportunity to ask questions concerning the experiment or the procedures involved
- Be instructed that consent to participate in the medical experiment may be withdrawn at any time and the subject may discontinue participation without prejudice
- Be given a copy of the signed and dated consent form
- And be given the opportunity to decide to consent or not to consent to a medical experiment without the intervention of any element or force, fraud, deceit, duress, coercion, or undue influence on the subject’s decision.

*YOUR SIGNATURE INDICATES THAT YOU HAVE READ AND UNDERSTAND THE ABOVE INFORMATION, THAT YOU HAVE DISCUSSED THIS STUDY WITH THE PERSON OBTAINING CONSENT, THAT YOU HAVE DECIDED TO PARTICIPATE BASED ON THE INFORMATION PROVIDED, AND THAT A COPY OF THIS FORM HAS BEEN GIVEN TO YOU.

_____________________________ _______________

Signature of Participant Date

***Person Obtaining Consent**

I attest that the requirements for informed consent for the medical research project described in this form have been satisfied – that the participant has been provided with the Experimental Subject’s Bill of Rights, if appropriate, that I have discussed the research project with the participant and explained to him or her in nontechnical terms all of the information contained in this informed consent form, including any risks and adverse reactions that may reasonably be expected to occur. I further certify that I encouraged the participant to ask questions and that all questions asked were answered.

_________________________________ _______________

Signature of Person Obtaining Consent Date
