# Supplementary material for: Human Adipose Derived Stromal Cells Heal Critical Size Mouse Calvarial Defects
Source: PLoS One. 2010 Jun 17;5(6):e11177. doi: 10.1371/journal.pone.0011177 (PMC2887361; doi:10.1371/journal.pone.0011177)
Supplement: Figure S3 — IRB Approval Letter. This IRB Approval Letter from the Stanford Institutional Review Board supports the harvest and study of human adipose derived stromal cells. (0.09 MB PDF) [file pone.0011177.s003.pdf]

STANFORD UNIVERSITY

Stanford, California 94305 - 5401

David Spiegel, M.D. (650) 725-5873
CHAIR, PANEL ON MEDICAL HUMAN SUBJECTS (650) 725-8013

Certification of Human Subjects Approvals

Date: September 25, 2009

To: Michael T. Longaker, MD, MBA, Surgery - Plastic and Reconstructive Surgery

Benjamin Levi MD, Sae Hee Ko MD, Annelise E Barron PhD, Anne M. Dubin M.D., Charles Kwok Fai Chan BS, Daniel Bernstein, Deepak Gupta MD, Daniel Meron Pinkas PhD, FangJun Jia PhD, Hansoo Park PhD, Howard Yuanhao Chang, Irving L. Weissman, Joseph Wu MD PhD, Michael Fischbein MD, Matthew B. Kerby PhD, Michelle Renee Marques BS, Ning Sun PhD, Philip E Oyer MD, Robert C Robbins MD, Sheng Ding B.S., M.S, Kitchener Daniel Wilson MD

From: David Spiegel, M.D., Administrative Panel on Human Subjects in Medical Research

Protocol Tissue Regeneration with Adipose Derived Mesenchymal Cells.------- -------- (SQL 96098)

Protocol ID: 2188 **IRB Number:** 350 (Panel: 3)

The IRB approved human subjects involvement in your research project on 09/25/2009. Prior to subject recruitment and enrollment, if this is: a Cancer-related study, you must obtain Cancer Center Scientific Review Committee (SRC) approval; a GCRC study, you must obtain GCRC approval; a VA study, you must obtain VA RD Committee approval; and if a contract is involved, it must be signed.

The expiration date of this approval is 03/09/2010 at Midnight. If this project is to continue beyond that date, you must submit an updated protocol in advance for the IRBs re-approval. If this protocol is used in conjunction with any other human use it must be re-approved. Proposed changes to approved research must be reviewed and approved prospectively by the IRB. No changes may be initiated without prior approval by the IRB, except where necessary to eliminate apparent immediate hazards to subjects. (Any such exceptions must be reported to the IRB within 10 working days.) Unanticipated problems involving risks to participants or others and other events or information, as defined and listed in the Report Form, must be submitted promptly to the IRB. (See Events and Information that Require Prompt Reporting to the IRB at http://humansubjects.stanford.edu.)

All continuing projects and activities must be reviewed and re-approved on or before Midnight of the expiration date. The approval period will be less than one year if so determined by the IRB. It is your responsibility to resubmit the project to the IRB for continuing review and to report the completion of the protocol to the IRB within 30 days.

Please remember that all data, including all signed consent form documents, must be retained for a minimum of three years past the completion of this research. Additional requirements may be imposed by your funding agency, your department, or other entities. (See Policy on Retention of and Access to Research Data at http://stanford.edu/dept/DoR/rph/2-10.html.)

This institution is in compliance with requirements for protection of human subjects, including 45 CFR 46, 21 CFR 50 and 56, and 38 CFR 16.

Includes: 1. New Grant for SPO # is 46480 for a new NIH stimulation bill
grant # 1RC2DE020771-01
PI: Longaker
Title: Calvarial Regeneration using Biomatrix-Encapsulated Skeletal Progenitors; ----

2. New Grant for SPO: 41258
grant # 3R21DE019274-02S1.
PI: Longaker
Title: The Enhanced Calvarial Regeneration Via RNAi-Mediated Suppression of BMP Antagonism
The funding section of the eprotocol application has also been updated;

Personnel Updates.

David Spiegel, M.D., Chair

Approval Period: 09/25/2009 THROUGH 03/09/2010

Review Type: EXPEDITED - MODIFICATION

Funding: NIH - SPO: 31444

Expedited Under Category: 5

Assurance Number: FWA00000935 (SU)

STANFORD UNIVERSITY

Stanford, California 94305 - 5401

David Spiegel, M.D. (650) 725-5873
CHAIR, PANEL ON MEDICAL HUMAN SUBJECTS (650) 725-8013

Certification of Human Subjects Approvals

Date: September 25, 2009

To: Michael T. Longaker, MD, MBA, Surgery - Plastic and Reconstructive Surgery

Benjamin Levi MD, Sae Hee Ko MD, Annelise E Barron PhD, Anne M. Dubin M.D., Charles Kwok Fai Chan BS, Daniel Bernstein, Deepak Gupta MD, Daniel Meron Pinkas PhD, FangJun Jia PhD, Hansoo Park PhD, Howard Yuanhao Chang, Irving L. Weissman, Joseph Wu MD PhD, Michael Fischbein MD, Matthew B. Kerby PhD, Michelle Renee Marques BS, Ning Sun PhD, Philip E Oyer MD, Robert C Robbins MD, Sheng Ding B.S., M.S, Kitchener Daniel Wilson MD

From: David Spiegel, M.D., Administrative Panel on Human Subjects in Medical Research

Protocol Tissue Regeneration with Adipose Derived Mesenchymal Cells.------- -------- (SQL 96098)

Protocol ID: 2188 **IRB Number:** 350 (Panel: 3)

The IRB approved human subjects involvement in your research project on 09/25/2009. Prior to subject recruitment and enrollment, if this is: a Cancer-related study, you must obtain Cancer Center Scientific Review Committee (SRC) approval; a GCRC study, you must obtain GCRC approval; a VA study, you must obtain VA RD Committee approval; and if a contract is involved, it must be signed.

The expiration date of this approval is 03/09/2010 at Midnight. If this project is to continue beyond that date, you must submit an updated protocol in advance for the IRBs re-approval. If this protocol is used in conjunction with any other human use it must be re-approved. Proposed changes to approved research must be reviewed and approved prospectively by the IRB. No changes may be initiated without prior approval by the IRB, except where necessary to eliminate apparent immediate hazards to subjects. (Any such exceptions must be reported to the IRB within 10 working days.) Unanticipated problems involving risks to participants or others and other events or information, as defined and listed in the Report Form, must be submitted promptly to the IRB. (See Events and Information that Require Prompt Reporting to the IRB at http://humansubjects.stanford.edu.)

All continuing projects and activities must be reviewed and re-approved on or before Midnight of the expiration date. The approval period will be less than one year if so determined by the IRB. It is your responsibility to resubmit the project to the IRB for continuing review and to report the completion of the protocol to the IRB within 30 days.

Please remember that all data, including all signed consent form documents, must be retained for a minimum of three years past the completion of this research. Additional requirements may be imposed by your funding agency, your department, or other entities. (See Policy on Retention of and Access to Research Data at http://stanford.edu/dept/DoR/rph/2-10.html.)

This institution is in compliance with requirements for protection of human subjects, including 45 CFR 46, 21 CFR 50 and 56, and 38 CFR 16.

Includes: 1. New Grant for SPO # is 46480 for a new NIH stimulation bill
grant # 1RC2DE020771-01
PI: Longaker
Title: Calvarial Regeneration using Biomatrix-Encapsulated Skeletal Progenitors; ----

2. New Grant for SPO: 41258
grant # 3R21DE019274-02S1.
PI: Longaker
Title: The Enhanced Calvarial Regeneration Via RNAi-Mediated Suppression of BMP Antagonism
The funding section of the eprotocol application has also been updated;

Personnel Updates.

David Spiegel, M.D., Chair

**Approval Period:**	09/25/2009 THROUGH 03/09/2010
**Review Type:**	EXPEDITED - MODIFICATION
**Funding:**	National Endowment for Plastic Surgery - SPO: 42387
**Expedited Under Category:**	5
**Assurance Number:**	FWA00000935 (SU)

LELAND STANFORD JUNIOR UNIVERSITY
DIE LUFT DER FREIHEIT WEHT
1891

STANFORD UNIVERSITY

Stanford, California 94305 - 5401

David Spiegel, M.D. (650) 725-5873

CHAIR, PANEL ON MEDICAL HUMAN SUBJECTS (650) 725-8013

Certification of Human Subjects Approvals

Date: September 25, 2009

To: Michael T. Longaker, MD, MBA, Surgery - Plastic and Reconstructive Surgery

Benjamin Levi MD, Sae Hee Ko MD, Annelise E Barron PhD, Anne M. Dubin M.D., Charles Kwok Fai Chan BS, Daniel Bernstein, Deepak Gupta MD, Daniel Meron Pinkas PhD, FangJun Jia PhD, Hansoo Park PhD, Howard Yuanhao Chang, Irving L. Weissman, Joseph Wu MD PhD, Michael Fischbein MD, Matthew B. Kerby PhD, Michelle Renee Marques BS, Ning Sun PhD, Philip E Oyer MD, Robert C Robbins MD, Sheng Ding B.S., M.S, Kitchener Daniel Wilson MD

From: David Spiegel, M.D., Administrative Panel on Human Subjects in Medical Research

Protocol Tissue Regeneration with Adipose Derived Mesenchymal Cells.------- -------- (SQL 96098)

Protocol ID: 2188 **IRB Number:** 350 (Panel: 3)

The IRB approved human subjects involvement in your research project on 09/25/2009. Prior to subject recruitment and enrollment, if this is: a Cancer-related study, you must obtain Cancer Center Scientific Review Committee (SRC) approval; a GCRC study, you must obtain GCRC approval; a VA study, you must obtain VA RD Committee approval; and if a contract is involved, it must be signed.

The expiration date of this approval is 03/09/2010 at Midnight. If this project is to continue beyond that date, you must submit an updated protocol in advance for the IRBs re-approval. If this protocol is used in conjunction with any other human use it must be re-approved. Proposed changes to approved research must be reviewed and approved prospectively by the IRB. No changes may be initiated without prior approval by the IRB, except where necessary to eliminate apparent immediate hazards to subjects. (Any such exceptions must be reported to the IRB within 10 working days.) Unanticipated problems involving risks to participants or others and other events or information, as defined and listed in the Report Form, must be submitted promptly to the IRB. (See Events and Information that Require Prompt Reporting to the IRB at http://humansubjects.stanford.edu.)

All continuing projects and activities must be reviewed and re-approved on or before Midnight of the expiration date. The approval period will be less than one year if so determined by the IRB. It is your responsibility to resubmit the project to the IRB for continuing review and to report the completion of the protocol to the IRB within 30 days.

Please remember that all data, including all signed consent form documents, must be retained for a minimum of three years past the completion of this research. Additional requirements may be imposed by your funding agency, your department, or other entities. (See Policy on Retention of and Access to Research Data at http://stanford.edu/dept/DoR/rph/2-10.html.)

This institution is in compliance with requirements for protection of human subjects, including 45 CFR 46, 21 CFR 50 and 56, and 38 CFR 16.

Includes: 1. New Grant for SPO # is 46480 for a new NIH stimulation bill
grant # 1RC2DE020771-01
PI: Longaker
Title: Calvarial Regeneration using Biomatrix-Encapsulated Skeletal Progenitors; ----

2. New Grant for SPO: 41258
grant # 3R21DE019274-02S1.
PI: Longaker
Title: The Enhanced Calvarial Regeneration Via RNAi-Mediated Suppression of BMP Antagonism
The funding section of the eprotocol application has also been updated;

Personnel Updates.

David Spiegel, M.D., Chair

Approval Period: 09/25/2009 THROUGH 03/09/2010
Review Type: EXPEDITED - MODIFICATION
Funding: NIH - SPO: 46480
Expedited Under Category: 5
Assurance Number: FWA00000935 (SU)

STANFORD UNIVERSITY

Stanford, California 94305 - 5401

David Spiegel, M.D. (650) 725-5873

CHAIR, PANEL ON MEDICAL HUMAN SUBJECTS (650) 725-8013

Certification of Human Subjects Approvals

Date: September 25, 2009

To: Michael T. Longaker, MD, MBA, Surgery - Plastic and Reconstructive Surgery

Benjamin Levi MD, Sae Hee Ko MD, Annelise E Barron PhD, Anne M. Dubin M.D., Charles Kwok Fai Chan BS, Daniel Bernstein, Deepak Gupta MD, Daniel Meron Pinkas PhD, FangJun Jia PhD, Hansoo Park PhD, Howard Yuanhao Chang, Irving L. Weissman, Joseph Wu MD PhD, Michael Fischbein MD, Matthew B. Kerby PhD, Michelle Renee Marques BS, Ning Sun PhD, Philip E Oyer MD, Robert C Robbins MD, Sheng Ding B.S., M.S, Kitchener Daniel Wilson MD

From: David Spiegel, M.D., Administrative Panel on Human Subjects in Medical Research

Protocol Tissue Regeneration with Adipose Derived Mesenchymal Cells.------- -------- (SQL 96098)

Protocol ID: 2188 **IRB Number:** 350 (Panel: 3)

The IRB approved human subjects involvement in your research project on 09/25/2009. Prior to subject recruitment and enrollment, if this is: a Cancer-related study, you must obtain Cancer Center Scientific Review Committee (SRC) approval; a GCRC study, you must obtain GCRC approval; a VA study, you must obtain VA RD Committee approval; and if a contract is involved, it must be signed.

The expiration date of this approval is 03/09/2010 at Midnight. If this project is to continue beyond that date, you must submit an updated protocol in advance for the IRBs re-approval. If this protocol is used in conjunction with any other human use it must be re-approved. Proposed changes to approved research must be reviewed and approved prospectively by the IRB. No changes may be initiated without prior approval by the IRB, except where necessary to eliminate apparent immediate hazards to subjects. (Any such exceptions must be reported to the IRB within 10 working days.) Unanticipated problems involving risks to participants or others and other events or information, as defined and listed in the Report Form, must be submitted promptly to the IRB. (See Events and Information that Require Prompt Reporting to the IRB at http://humansubjects.stanford.edu.)

All continuing projects and activities must be reviewed and re-approved on or before Midnight of the expiration date. The approval period will be less than one year if so determined by the IRB. It is your responsibility to resubmit the project to the IRB for continuing review and to report the completion of the protocol to the IRB within 30 days.

Please remember that all data, including all signed consent form documents, must be retained for a minimum of three years past the completion of this research. Additional requirements may be imposed by your funding agency, your department, or other entities. (See Policy on Retention of and Access to Research Data at http://stanford.edu/dept/DoR/rph/2-10.html.)

This institution is in compliance with requirements for protection of human subjects, including 45 CFR 46, 21 CFR 50 and 56, and 38 CFR 16.

Includes: 1. New Grant for SPO # is 46480 for a new NIH stimulation bill
grant # 1RC2DE020771-01
PI: Longaker
Title: Calvarial Regeneration using Biomatrix-Encapsulated Skeletal Progenitors; ----

2. New Grant for SPO: 41258
grant # 3R21DE019274-02S1.
PI: Longaker
Title: The Enhanced Calvarial Regeneration Via RNAi-Mediated Suppression of BMP Antagonism
The funding section of the eprotocol application has also been updated;

Personnel Updates.

David Spiegel, M.D., Chair

Approval Period: 09/25/2009 THROUGH 03/09/2010

Review Type: EXPEDITED - MODIFICATION

Funding: NIH - SPO: 41258

Expedited Under Category: 5

Assurance Number: FWA00000935 (SU)

LELAND STANFORD JUNIOR UNIVERSITY
DIE LUFT DER FREIHEIT WEHT
1891
